# Taxonomically Restricted Wheat Genes Interact With Small Secreted Fungal Proteins and Enhance Resistance to Septoria Tritici Blotch Disease

**DOI:** 10.3389/fpls.2020.00433

**Published:** 2020-05-07

**Authors:** Ciarán J. Brennan, Binbin Zhou, Harriet R. Benbow, Sobia Ajaz, Sujit J. Karki, James Gerard Hehir, Aoife O’Driscoll, Angela Feechan, Ewen Mullins, Fiona M. Doohan

**Affiliations:** ^1^UCD School of Biology and Environmental Science and UCD Earth Institute, UCD O’Brien Centre for Science (East), University College Dublin, Belfield, Ireland; ^2^School of Agriculture and Food Science, University College Dublin, Belfield, Ireland; ^3^Department of Crop Science, Teagasc, Carlow, Ireland

**Keywords:** *Zymoseptoria tritici*, Orphan gene, Small secreted proteins, Effectors, wheat, STB

## Abstract

Understanding the nuances of host/pathogen interactions are paramount if we wish to effectively control cereal diseases. In the case of the wheat/*Zymoseptoria tritici* interaction that leads to Septoria tritici blotch (STB) disease, a 10,000-year-old conflict has led to considerable armaments being developed on both sides which are not reflected in conventional model systems. Taxonomically restricted genes (TRGs) have evolved in wheat to better allow it to cope with stress caused by fungal pathogens, and *Z. tritici* has evolved specialized effectors which allow it to manipulate its’ host. A microarray focused on the latent phase response of a resistant wheat cultivar (cv. Stigg) and susceptible wheat cultivar (cv. Gallant) to *Z. tritici* infection was mined for TRGs within the Poaceae. From this analysis, we identified two TRGs that were significantly upregulated in response to *Z. tritici* infection, *Septoria-responsive TRG6* and *7 (TaSRTRG6 and TaSRTRG7)*. Virus induced silencing of these genes resulted in an increased susceptibility to STB disease in cvs. Gallant and Stigg, and significantly so in the latter (2.5-fold increase in STB disease). *In silico* and localization studies categorized TaSRTRG6 as a secreted protein and TaSRTRG7 as an intracellular protein. Yeast two-hybrid analysis and biofluorescent complementation studies demonstrated that both TaSRTRG6 and TaSRTRG7 can interact with small proteins secreted by *Z. tritici* (potential effector candidates). Thus we conclude that TRGs are an important part of the wheat-*Z. tritici* co-evolution story and potential candidates for modulating STB resistance.

## Introduction

Septoria tritici blotch (STB) is a foliar disease of wheat caused by the haploid, pathogenic fungus *Zymoseptoria tritici* (formally known as *Mycosphaerella graminicola*; anamorph: *Septoria tritici*). It is a foliar disease that affects wheat crops worldwide: it is the primary foliar disease of winter wheat in the majority of countries within Western Europe, including Ireland (Lynch et al., 2017). Disease-associated yield losses are a consequence of the reduced photosynthetic area of the host. Severe epidemics of STB have historically been reported to cause yield losses of up to 50% (Eyal, 1987). However, more contemporary estimates in the United Kingdom have placed losses closer to 20% when STB resistant varieties are used in conjunction with a viable fungicide management plan (Fones and Gurr, 2015). STB is a polycyclic disease capable of completing its lifecycle up to six times per season (assuming the formation of sexual pynidiospores takes 20 days from spore germination) (Fones and Gurr, 2015). A hallmark of *Z. tritici* infection in wheat is the prolonged asymptomatic latent phase that occurs after infection takes place. This latent phase varies dramatically between cultivars, inoculum and environmental conditions Prolonging this phase further can help to mitigate the effects of STB due to prolonged photosynthesis and a reduction in fungal inoculum (Hehir et al., 2017).

Infection of wheat by *Z. tritici* can be termed either compatible or incompatible, the former resulting in successful colonization, while the latter involves rapid recognition of the infection and the prompt activation of host defenses (Adhikari et al., 2007). Over the last 10 years, studies have focused on deciphering the mechanisms underpinning the response of wheat to STB disease, including the susceptible and resistance responses (Ray et al., 2003; Shetty et al., 2003, 2007, 2009; Adhikari et al., 2007; Keon et al., 2007; Rudd et al., 2008). The activation of a HR-like response through a mitogen-associated protein kinase (MAPK) cascade shows that the compatible *Z. tritici*-wheat interaction shares similarities with an incompatible host-biotroph interaction (Rudd et al., 2008). The burgeoning field of fungal effector/small secreted protein biology has given us new insights into the nuances of the wheat/*Z. tritici* interaction allowing for the investigation of interactions in both the host (Marshall et al., 2011) and model non-hosts (Kettles et al., 2016). Recently, a wall-associated receptor kinase-like protein termed Stb6 was cloned and its interaction with a small cysteine-rich fungal effector protein (AvrStb6) demonstrated a gene-for-gene resistance to *Z. tritici* (Zhong et al., 2017; Kema et al., 2018; Saintenac et al., 2018). While this was the first major STB resistance gene to be cloned, race and non-race -specific resistance to *Z. tritici* has been characterized in wheat genotypes, and other major resistance loci have been identified and mapped to the hexaploid wheat genome (Orton et al., 2011).

The recently completed sequencing of the wheat genome (Consortium, 2018), together with the sequenced *Z. tritici* genomes (Goodwin et al., 2011; Badet et al., 2019), now provides us with the tools necessary to better characterize more of the protein-protein interactions unique to this host/pathogen combination, including the study of the co-evolution of wheat and *Z. tritici*. Proteins can evolve to enable organisms to tolerate or overcome specific biotic or abiotic stresses associated with, or unique to, their ecological niche (Challabathula and Bartels, 2013; Luhua et al., 2013; Yadeta et al., 2014). Taxonomically restricted genes (TRGs) are a broad classification of genes that exist in a limited number of closely related organisms. Based on the period through which an orphan’s evolution occurred, coupled with the homology to closely related species, such a gene can be classified as a TRG, a taxon-specific gene or a species-specific gene. Depending on how origination occurs, remnant domains may be present within TRGs arising through gene fusion or gene duplication (Buljan et al., 2010). Indeed, strict definitions for their delineation of TRGs based on nucleotide or protein similarity have not been agreed upon, and vary depending on individual studies (Lin et al., 2010; Donoghue et al., 2011). “Orphan gene” is an umbrella term used to describe rapidly evolving genes that are restricted to a specifically defined grouping of organisms and do not contain significant homology to any other organisms outside of this grouping (Wissler et al., 2013). TRGs can arise though a variety of mechanisms such as gene fusion, gene fission, exon shuffling, retroposition, horizontal gene transfer and *de novo* mutation/origination. *De novo* mutation/origination is a mechanism in which new genes arise from a gene framework normally restricted to intron regions, capable of forming proto-genes but these are only transcribed in times of stress to give potential advantages (Carvunis et al., 2012). Genes arising from *de novo* mutations are thought to evolve as simple genes and gradually become more complex over time (Siepel, 2009). Next generation sequencing will help close the gap in our knowledge of the gene incidence and diversity of TRGs. Compared to older genes, the expression of TRGs tends to be less ubiquitous than that of their conserved counterparts, often restricted to specific tissue (Donoghue et al., 2011) and boasting lower expression (Carvunis et al., 2012). Thus, they can be easily overlooked.

TRGs have been studied in terms of their role in the evolution and virulence of *Z. tritici*. In a study comparing the orphanome of two *Z. tritici* isolates, TRGs were found to be significantly enriched in regions coding for secreted effector proteins (Plissonneau et al., 2016); the findings seem to lend credence to the concept that TRGs play a crucial role in maintaining successful pathogenesis. Recently, it was demonstrated that rapidly evolving genes of *Z. tritici* are central to both virulence and host specialization. Removal and replacement of these genes with orthologs from closely related *Z. pseudotritici* and *Z. ardabiliae* which were still being governed by the *Z. tritici* native promotor did not restore the virulence, further demonstrating the importance of these TRGs in the pathogens specialization (Poppe et al., 2015). Serval pathogen-responsive TRGs have been observed in model, ornamental and agricultural plants alike which bolster the defensive properties of the host (Xiao et al., 2009; Mandal et al., 2013; Yadeta et al., 2014) and it is thus likely that TRGs play a role in the wheat response to STB disease. Evidence from the wheat/*Fusarium* pathosystem demonstrated the role of TRGs in disease resistance. The Pooideae*-*restricted gene *TaFROG* (*Triticum aestivum* Fusarium Resistance Orphan Gene) was shown to enhance wheat resistance to the *Fusarium* mycotoxin and virulence factor deoxynivalenol (DON) and it also enhanced resistance to Fusarium head blight (FHB) caused by *Fusarium graminearum.* TaFROG interacts with proteins of diverse cellular function, including the evolutionarily conserved SnRK1 (Sucrose-non-Fermenting Kinase) subfamily, illustrating TRG integration into conserved signaling processes (Perochon et al., 2015, 2019).

The hypothesis of this study was test if taxonomically restricted genes play a role in host resistance to STB. To test this, existing microarray data that examined the expression profiles of two wheat cultivars, differentially responsive to STB, was mined for *Z. tritici*-responsive, taxonomically restricted wheat genes. Two *Z. tritici*-response taxonomically restricted genes were characterized in terms of their response to, and effects on, the susceptibility of wheat to STB disease. Thereafter, their ability to interact with small secreted *Z. tritici* proteins was investigated. Based on the results, the positive role of TRGs in wheat defense against STB is discussed.

## Materials and Methods

### Plant and Fungal Material

Winter wheat cultivars (cvs) Stigg [Limagrain; disease rating = 8 (ADHB, 2012)] and Gallant [Syngenta; disease rating = 4 (AHDB, 2016)], were selected for this study, based on previous performances in both the field and glasshouse (Hehir et al., 2017). **Zymoseptoria tritici** isolate IPO323 was kindly provided by Dr. Gert Kema (Wageningen University, Netherlands) and maintained in glycerol stocks at −80°C. **Zymoseptoria tritici** isolate Cork Cordiale 4 and 560.11 were kindly provided by Drs Stephen Kildea and Thomas Welch of Teagasc Crops research, Co. Carlow. Fungal spores of **Zymoseptoria tritici** isolate IPO323, Cork Coridale 4 and 560.11 were harvested from 7-day-old cultures on potato dextrose agar plates (Oxoid, United Kingdom) were used in all host-pathogen interaction experiments.

### STB Seedling Experiments

All STB infection experiments were carried out as described by Hehir et al. (2017), with the modifications as follows. Seeds of cvs. Stigg and Gallant were grown until an early growth stage (see below), at which point the second leaf was treated until run-off with either a pycnidiospore suspension of *Z. tritici* strain IPO323 (1 × 10^6^ ml^–1^ with 0.2% Tween20) or 0.2% Tween20 (control plants). For the analysis of the wheat x isolate IPO323 interaction, two experiments were conducted which collectively assessed disease development and the transcriptome (microarray) response of cvs. Stigg and Gallant. Both contained three independent trials. In the first experiment, the second leaf was inoculated at growth stage (GS)14 (Zadocks et al., 1974) and disease was scored at 16, 18, 20, 22, 24, 26, and 28 dpi (10 plants/biological replicates per genotype per treatment were assessed at each time point in each trial). Disease was assessed as% diseased leaf area bearing pycnidia and latent period. Latent period was calculated as days from inoculation to first appearance of lesions bearing pycnidia. The area under the disease progress curve (AUDPC) was calculated as described by Shaner and Finney (1977). For microarray analysis, in each of the three trials, the second leaf of plants was inoculated at GS12 and harvested at timepoints 4, 8 and 12 dpi, covering the asymptomatic (i.e., latent phase) and early necrotrophic phases of the *Z. tritici* infection cycle. Within each trial 5 leaves were treated per genotype per timepoint and these were used to form one composite bulk (resulting in three biological replicates per treatment per time point), plus additional plants were grown to verify that the disease progressed as expected in the microarray experiment. While the initial microarray and latent phase duration experiments were conducted with the common laboratory *Z. tritici* isolate IPO323, the latter silencing and time course experiments were conducted with the more aggressive, contemporary *Z. tritici* field isolate Cork Cordiale 4. For the analysis of the wheat x isolate Cork Cordiale 4 interaction, an independent STB timecourse of cvs. Stigg and Gallant was performed comprising three independent trials. In each trial, the third leaf of plants was inoculated at GS 21 and plants were harvested at 4, 8, or 12 dpi (4 plants per treatment per time point per genotype). Due to the prolonged latent phase of the pathogen and the natural senescence that occurs in the second leaf in wheat seedlings, the third leaf was selected for infection at a latter growth stage to reduce the possibility of natural chlorosis accelerating disease progression. Within each trial, replicate samples were used to yield one composite bulk per treatment which was subsampled twice for RNA extraction (resulting in 3 biological × 2 technical replicates per treatment per time point).

### RNA Extraction

Total RNA was extracted from frozen leaf tissue using either the Tri-reagent procedure (Sigma-Aldrich), or the RNeasy Plant Mini kit (Qiagen), following the manufacturer protocols. Samples were DNase-treated using the Qiagen DNAse kit according to the manufacturer’s protocol. Leaf tissue samples were desiccated in open 2 ml Eppendorf safe seal tubes for 48 hours using a FreeZone12 vacuum freezer drier (Labconco, United States) and stored at −80°C. Two 3 mm sterile and RNAseZap (Thermo Fisher Scientific, United States) treated tungsten ball bearings were added to the samples and these were homogenized in a TissueLyser II (Qiagen, Germany) at 45 Hz for 60 s. To test if all genomic DNA had been removed from the RNA sample, each RNA sample was subjected to qPCR analysis of *TaGAPDH2* (Supplementary Table S1 and below for qPCR methodology).

### Microarray Analysis

Transcriptional analysis of a resistant (cv. Stigg) versus that of a susceptible (cv. Gallant) response to *Z. tritici* was carried out using the Affymetrix 61K Wheat GeneChip^®^ Array^1^. RNA samples were sent on dry ice to ATLAS Biolabs, Ltd. (Berlin, Germany) where the quality and the quantity of each RNA sample was determined using an Agilent 2100 Bioanalyzer and RNA 6000 Pico Series II Chips (Agilent). Microarray data was analyzed by ATLAS Biolabs. The data were normalized using log2 transformation per chip and per probe. Differential expression analysis was conducted per timepoint per cultivar and *P-*values were corrected for false discovery (FDR) using the BH method (Benjamini and Hochberg, 1995). Statistical analysis using student *t*-tests were performed to generate significant probes with a fold-change ≥ 2 and FDR-adjusted *P* ≤ 0.05 between “control” and “treated” samples. The plant expression database (PLEXdb)^2^ (Dash et al., 2012) was used to acquire the probe sequence for the 61K Affymetrix microarray GeneChip data used in this study. IWGSC Refseq version 1.1 cDNA annotation (IWGSC, 2018) was accessed from the IWGSC URGI portal^3^ and a local BLASTn database of the Refseq was created with the “makeblastdb” command from BLAST + (Camacho et al., 2009). Probe sequences were BLASTn searched against this database using default parameters and the top hits for each Affymetrix probe was filtered. As the Affymetrix probe sequences are not homoeolog-specific (i.e., each probe sequence may align to multiple homoeologs and paralogs of a gene family), all homoeologs/paralogs of a gene with high homology to a probe sequence were considered to be represented by that probe sequence.

### Mining for Taxonomically-Restricted Genes and Selection of Candidate Genes

The corresponding IWGSC sequences for every differentially expressed probe were BLASTx queried against the NCBI non-redundant protein database^4^ to identify their taxonomic distribution. Thresholds of *E* ≤ 1e^–5^ and percentage identity ≥ 50% were used to classify genes based on their similarity to genes from other species. Genes with a hit to a species outside of the Poaceae were not considered taxonomically restricted. Genes with no hit to a species outside of the Poaceae were designated as Poaceae-specific taxonomically-restricted genes. The amino acid sequences for the BLASTx top hit from each species with a significant hit were retrieved from NCBI, and a multiple sequence alignment was created using the Clustal Omega function in the R package “msa” (Bodenhofer et al., 2015). Distance matrices were created from the alignments and a neighbor-joining phylogenetic trees were constructed with the R package “ape.” Phylogenetic trees were visualized in Figtree v1.4.4 (accessed November 2019)^5^. Candidate genes were further prioritized for validation and characterization based on their responsiveness to *Z. tritici* during the latent phase of infection, their higher expression in the resistant cultivar both relative to the control treatment and the susceptible cultivar and their ability to be cloned into silencing constructs and be quantified using qRT-PCR. Select taxonomically-restricted candidate genes were surveyed for pathogen-responsiveness against publicly available transcriptomics data for *Z. tritici* (Yang et al., 2013), *Blumeria graminis* (Zhang et al., 2014), *Fusarium graminearum* (Kugler et al., 2013), *Fusarium pseudograminearum* (Powell et al., 2017) and the PAMP elicitors chitin and flagellin 22 (flg22) (Ramirez-gonzalez et al., 2018), available on http://www.wheat-expression.com/(accessed 2019) (Borrill et al., 2016), previously reanalyzed for differential expression by Benbow et al. (2019).

### cDNA Synthesis and qRT-PCR Analysis

For qRT-PCR, cDNA synthesis was performed using Oligo(dT)_12__–__18_ Primer, M-MLV Reverse Transcriptase and RNaseOUT^TM^ Kits (Invitrogen, United States) in accordance with the manufacturer’s protocol. cDNA synthesis was validated by PCR using *TaGAPDH2* primers (Supplementary Table S1). *TaSRTRG* qRT-PCR primers were designed to target identical sequence regions across all 3 homeologs of each gene at the 3′ end. Primers were designed using online Primer3 software^6^. Primers were tested using cDNA from *Z. tritici* treated wheat tissue from cv. Stigg at 12 DPI (Supplementary Table S1). All gene-specific primers were assessed for their individual efficiencies and melt curves in accordance with the MIQE standards (Bustin et al., 2009). The thermal cycle for all genes consisted of: initial heat for 10 s at 95°C, 40 cycles of 5 s at 95°C, 30 s at 60°C, 1 cycle of 1 min at 95°C, 81 cycles of 30 s at 55°C. Ct values were calculated using the average values of each treatment/timepoint/cDNA combination. Two independent qRT-PCR analysis were performed per sample and results were analyzed using the ΔΔCt method [ΔCt(treated) - ΔCt(mock)]. Samples were run in conjunction with two reference genes (alpha tubulin and GAPDH2; Supplementary Table S1). Normalized target gene expression was calculated using the formula 2^–ΔΔCt^ (Livak and Schmittgen, 2001).

### Virus-Induced Gene Silencing

Virus-induced gene silencing (VIGS) was used to determine the impact of TaSRTRG genes on STB disease, based on the coat-protein modified *Barley stripe mosaic virus* (BSMV) method (Holzberg et al., 2002; Scofield et al., 2005; Gunupuru et al., 2019). VIGS constructs and primers were designed to target all three homoeologs of each *TaSRTRG* gene (Supplementary Table S2). Due to the small size of the genes, only a single construct was designed for each gene and primers used for qRT-PCR validation of VIGS were designed to amplify a region distal to the VIGS fragment. Fragments were amplified from cDNA generated from *Z. tritici*-treated wheat tissue from cv. Stigg at 12 dpi using the mix and thermal cycle outlined above. The α, β, and γ RNAs form the tripartite genome of BSMV and all fragments for silencing were cloned into the γ vector pSL038-1. Primers used for fragment cloning are detailed in Supplementary Table S1 and the method was previously described (Perochon et al., 2015). Inserts were confirmed by sequencing (Macrogen, Netherlands). The α and γ plasmids (containing all silencing fragments, the PDS positive control and empty vector) were linearized with *Mlu*I and NE buffer 3.1 (New England BioLabs, United States) in accordance with the manufacturer’s protocol. The β plasmid was linearized with *Spe*I and CutSmart^TM^ buffer (New England BioLabs, United States) in accordance with the manufacturer’s protocol. *In vitro* transcription of the linearizer plasmids was performed using a mMESSAGE mMACHINE^®^ T7 Kit (Ambion, United States) in accordance with the manufacturer’s protocol and successful transcription was confirmed by gel electrophoresis. Successful *in vitro* transcripts were stored at −80°C to prevent degradation. Four BSMV silencing constructs were used in this study. *TaSRTRG* silencing constructs were designated BSMV:*TaSRTRG6* and BSMV:*TaSRTRG7*. Two BSMV controls were also used during this study: an empty vector BSMV construct referred to as BSMV:00 and a PDS positive control simply referred to as PDS. For the VIGS experiment, three independent trials were conducted, each containing four biological replicates per cultivar per treatment. Viral transcripts of α, β, and γ (or modified γ) RNAs were mixed with FES buffer (0.1 M glycine, 0.06 M K_2_HPO_4_, 1% w/v tetrasodium pyrophosphate, 1% w/v bentonite, 1% w/v celite, pH 8.5) and applied in equal concentrations (1:1:1) as described in Gunupuru et al. (2015). Two-liter posts containing 4 seedlings per pot were grown in John Innes Compost No. 2 (Westland Horticulture, United Kingdom) for growing on until the desired growth stage was achieved. Plants were grown under controlled conditions using 6x Philips Master TL-D 36W/840 cold fluorescent bulbs (Philips, Netherlands) consistently emitting a combined 12,000 lux over a 15/9 light/dark cycle at 80% relative humidity at 19°C. Transcript mixtures were applied to a fully extended 2nd leaf of wheat seedlings using the thumb and index finger of a gloved hand and gently rubbing pinched fingers the length of the leaf twice. Plants were placed in low light conditions overnight to aid in recovery from mechanical stress and then returned to normal growth conditions for 7 days before *Z. tritici* inoculation. *Z. tritici* was spray-inoculated onto both the 3rd and 4th leaves using a pycnidiospore suspension of *Z. tritici* strain Cork Cordiale 4 (1 × 10^6^ ml^–1^ with 0.2% Tween20) or 0.2% Tween20 (control plants). The 3rd leaf of each plant was harvested 4 days post inoculation (dpi) and used for qRT-PCR-based validation of gene silencing. RNA was extracted from each treated leaf used for qRT-PCR analysis. The 4th leaf from each treated plant was used for phenotyping: disease symptoms were visually assessed for percentage leaf area bearing pycnidia on the 4th leaf at 28 dpi with *Z. tritici*.

#### Cloning of *TaSRTRG* Genes, *Z. tritici* SSP and Plasmid Construction

The full-length of *TaSRTRG6, TaSRTRG7* and twenty-seven *Z. tritici* candidate SSPs (Karki, unpublished) were amplified from the first strand cDNA synthesized form total RNA derived from a *Z. tritici* isolate IPO560 or Cork Cordiale 4 infected wheat leaf (cv. Stigg). The specific primers with attB1 and attB2 sites were used in amplification. Gateway PCR products flanked by the attB1 and attB2 sites were recombined into the pDONR 207 vector (Invitrogen, United States) to create the corresponding entry clones with attL1 and attL2 sites following the manufacturer’s protocol. The relevant entry clones (pENTR) were subsequently recombined into appropriate destination vectors via an LR reaction to create the expression constructs. All primers used are shown in Supplementary Table S1.

#### Protein Secretion via Yeast Expression System

Secretion of the TaSRTRG proteins was predicted using SignalP v. 5.0^7^ (Armenteros et al., 2019). To test protein secretion, a yeast expression system was employed, which was based on vector pGADT7. This vector carries a truncated invertase gene, *SUC2*, without a signal peptide, amplified from yeast strains BY4741. A linker (HA tag-Kex2 cleavage site) was added between the Gateway Reading Frame Cassette and the truncated *SUC2* gene. This Kex2 cleavage site improves efficacy of yeast secretion and avoids any negative effects of the fusion protein on SUC2 activity. To construct the invertase negative yeast strain, the full length *SUC2* gene of yeast strain SEY6210 was knocked out by homologous recombination. The pGAD-derived plasmids were transformed into the invertase negative yeast strain individually. Positive transformants were assayed on a synthetic dropout plates with sucrose as carbon supplies respectively after growing 3–4 days at 28°C. Yeast spotting on the media was performed using serial dilutions from an initial OD600 of 0.1, 0.01, and 0.001, respectively. Three biological replicates were included per construct in each of three trials.

### Yeast Two-Hybrid Analysis of Interactions Between TaSRTRG Genes and *Z. tritici* SSPs

To test TaSRTRG interactions with *Z. tritici* SSPs, relevant constructs of pENTER-ΔSP:*TaTaSRTRG6* (lacking signal peptide) pENTER-*TaTaSRTRG7*; pENTER-ΔSP:SSP (lacking signal peptide) were recombined into the yeast bait and prey vectors derived from pGADT7 and pGBKT7 of the Two-Hybrid System with Gateway Technology. The bait and prey vectors were transformed into a yeast strain (Y2H Matchmaker Gold, Clontech, United States) and grown in solid minimal Synthetic Defined media (SD) lacking both leucine and tryptophan (SD/-Trp/-Leu) for 3 days at 28°C. The double transformants yeast cells were dropped in SD/-Trp/-Leu/-His (TLH) and SD/-Trp/-Leu/-His/-Ade (TLHA) then grown at 28°C for 3–7 days. Three biological replicates were conducted per trial per construct combination and analysis of protein-protein interactions were performed as described previously by Perochon et al. (2015).

### Subcellular Localization and Bimolecular Fluorescence Complementation (BiFC)

*Nicotiana benthamiana* seeds were synchronized for 3 days at 4°C in a cold room. Seeds were subsequently grown as described (Perochon et al., 2015) at 19°C with a 16/8 light/dark for 5 weeks before infiltration for all experiments. For the subcellular localization, the expression vectors pEAQ-HT:TaSRTRG 7:YFP and pAM-PAT-35S:CFP were co-expressed in the leaves of *N. benthamiana* via an *Agrobacterium-*mediated transient expression system, used as described previously (Perochon et al., 2015). Three independent transformations per construct combination were analyzed in each of three trials (*n* = 9 per construct combination). The cyan fluorescence protein (CFP) and yellow fluorescence protein (YFP) fluorescence in *N. benthamiana* leaves was analyzed with an Olympus fluoview FV1000 Confocal Laser Scanning Microscope (Olympus, Japan) 2–3 days after infiltration. YFP fluorescence was excited with the 515 nm wavelength and detected in the range between 530 and 630 nm. CFP fluorescence was excited with the 405 nm wavelength and recorded in one of the confocal channels in the 460–500 nm emission range.

Bimolecular fluorescence complementation (BIFC) was used to test protein-protein interactions *in planta*. The relevant entry clones (pENTR) of ΔSP:*TaSRTRG6*, *TaSRTRG7*, ΔSP:SSPss were recombined into BiFC vectors pDEST-VYCE^GW^ and pDEST-VYNE^GW^ (Gehl et al., 2009). This generated constructs wherein proteins were fused to the YFP C-terminal (YFP^C^) or N-terminal fragment (YFP^N^). The vectors were then transformed into the *A. tumefaciens* strain GV3101. Co-infiltration of *A. tumefaciens* strains harboring the BiFC constructs and the p19 silencing plasmid was carried out at a final OD_600_ = 0.5:0.5:0.1. Three independent transformations per construct combination were analyzed in each of three trials (*n* = 9 per construct combination). Epidermal cells of tobacco leaves were assayed for YFP fluorescence 2–3 days after infiltration using the same Confocal Laser Scanning Microscope and Excitation/emission range as described above.

### Expression of *Z. tritici* SSPs in *N. benthamiana*

The expression vector pEAQ-HT-DEST3 containing the *Z. tritici* SSP genes were transformed into *Agrobacterium tumefaciens* strains GV3101 (Perochon et al., 2015) by electroporation at a voltage of 1.44 kV for 5 ms. The relevant GV3101 strains were grown in LB liquid medium containing gentamicin (20 μg/ml), kanamycin (50 μg/ml), and rifampicin (50 μg/ml) at 28°C overnight. Bacteria were centrifuged at 4000 rpm for 10 min and washed once with distilled water. Bacterial cells were resuspended in infiltration buffer (10 mM MES pH 5.6, 10 mM MgCl_2_, 150 μM acetosyringone) to an OD_600_ = 1.0 and incubated in the dark for 2 h at room temperature. The leaves on 4–5 weeks old *N. benthamiana* plants (grown as described above) were infiltrated with a 1 ml needleless syringe. Cell death induction was determined by visual assessment relative to the control treatment. Three independent infiltrations per construct were analyzed in each of three trials (*n* = 9 per SSP).

### Protein Expression and Purification of TaSRTRG Proteins

The amplified Coding Sequence (CDS) of the *TaSRTRG* genes were purified and then digested with *Bam*HI and *Xho*I, which were ligated into the expression vector pET-45b(+). The recombinant plasmids were verified by sequencing (Macrogen, Netherlands) then transformed into *E. coli* strain BL21 (DE3). The recombinant strains were firstly cultured in 4 mL of LB medium (50 mg/mL Carbenicillin) overnight at 37°C. 1 mL of the overnight culture was transferred into 400 mL of LB medium and grown to an OD_600_ = 0.4–0.6. IPTG was added to 0.25 mM and allowed to induce for 20 h at 25°C. Bacteria were harvested and kept in a REVCO EXF −80°C freezer (Thermo Fisher Scientific, United States) for purification. The proteins were purified using HisTrap FF 1 mL column (GE Healthcare, United Kingdom) and AKTAprime system (GE Healthcare, United Kingdom). The proteins were collected and analyzed by SDS-PAGE under denaturing conditions. Some aliquots were desalted using PD-10 Desalting Columns (GE Healthcare, United Kingdom) following the manufacturer’s instructions.

### Inhibitory Activity Assay of TaSRTRG Proteins

The TaSRTRG inhibitory activities against trypsin T4799 (Sigma-Aldrich, France) and chymotrypsin C4129 (Sigma-Aldrich, France) were determined by incubating purified TaSRTRG proteins together with the enzymes and measuring the change in absorbance at 25°C for 30 min. The remaining trypsin activity was measured with TAME T4626 (Sigma-Aldrich, France) as a substrate and the changes in absorbance at 247 nm were measured with a UV-Visible spectrometer SPECTROstar Nano Microplate Reader (BMG LABTECH, Germany) in a 0.3 ml reaction mixture containing 46 mM Tris/HCl (pH 8.1), 11.5 mM CaCl2 and 1 mM TAME. For the measurement of a-chymotrypsin activity, BTEE B6125 (Sigma-Aldrich, France) was used and the absorbency at 256 nm was followed by a 0.3 ml reaction mixture containing 40 mM Tris/HCl (pH 7.8), 50 mM CaCl2 and 0.5 mM BTEE. Commercial soybean BBI T9777 (Sigma-Aldrich, France) was assayed in parallel to serve as a positive control. Three independent assays were conducted per substrate per protein in each of three trials (*n* = 9 per SSP per substrate).

### Statistical Analysis

Data from the VIGS qRT-PCR and phenotyping were analyzed in SPSS v 24. Data were checked for correlation between trials and replicates using a Spearman’s rank correlation analysis. A Kolmogorov–Smirnov test for was used to test for normality and determine the distribution of the data. Transformation of the data to a normal distribution was unsuccessful so the data were analyzed using a Kruskal–Wallis test.

## Results

### Validation of the STB Resistance of cv. Stigg and the Susceptibility of cv. Gallant

**FIGURE 1:**
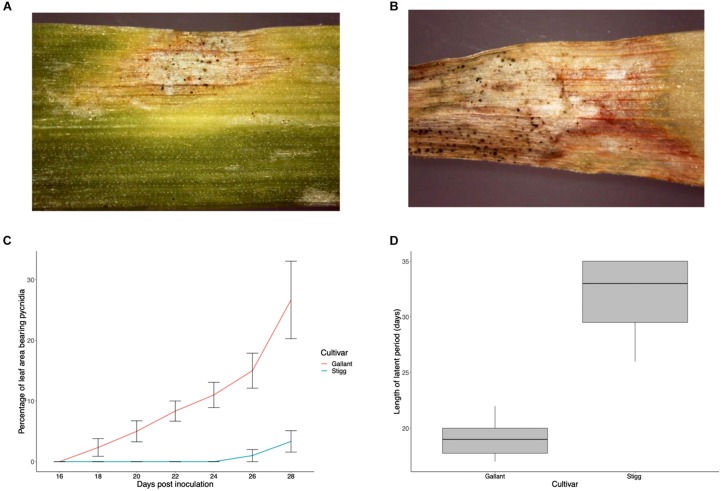
Development of Septoria tritici blotch disease on wheat cultivars Stigg and Gallant. **(A,B)** Representative photograph of cv. Stigg and cv. Gallant at 28 dpi. **(C)** Means of percentage necrotic areas bearing pycnidia for disease scores on the second leaf from day 17–28 dpi. **(D)** Boxplot of Latency period in cv, Gallant and Stigg. Latency period was calculated as days from inoculation to first appearance of lesions bearing pycnidia. If pycnidia were not detached on replicates by day 35, these samples were given a value of day 35 (10 biological replicates per time point per treatment per cultivar were measured per trial and 3 trials were conducted). Bars indicate SEM.

Glasshouse trials were carried out to validate that cvs. Stigg and Gallant displayed similar responses to *Z. tritici* isolate IPO323 as they did in the field (Hehir et al., 2017). Disease was assessed in terms of the rate of disease progression on the leaf (calculated as% leaf area with lesions bearing pycnidia from days 0 to 28 dpi) and latent period (calculated as days from inoculation to first appearance of lesions bearing pycnidia). Symptoms of STB on host leaves were first detected in the susceptible cultivar cv. Gallant at 10 dpi. Subsequently, the tissue turned necrotic and the pathogen started sporulating, with pycnidia visible by day 18. The latent phase of the cv. Stigg-*Z. tritici* interaction was significantly longer (32 days) as compared to cv. Gallant (19 days) (*P* < 0.001). This resulted in delayed progression of symptoms, with cv. Stigg only exhibiting <5% necrotic areas bearing pycnidia at 28 dpi compared to Gallant which had over 25% necrotic areas bearing pycnidia at this stage (Figure 1).

### Delineation of STB-Responsive, Taxonomically Restricted, Wheat Genes

Transcriptomic analysis was conducted using the Affymetrix 61K wheat GeneChip for cvs. Stigg and Gallant at three distinct timepoints (4, 8, and 12 dpi) covering the asymptomatic and early necrotrophic phases of the *Z. tritici* lifecycle. Differentially expressed genes (DEGs) were identified across all cultivars/time-points in response to *Z. tritici* (| log_2_ fold change| > 1, adjusted *P* < 0.01; Table 1 and Supplementary Table S3). At each time point, cv. Gallant had more STB up-regulated genes than cv. Stigg, and the same was true for down-regulated genes except at 8 dpi. At any given time-point, TRGs accounted for 0–6.5% of the up- or down-regulated DEGs (Table 1). TRG down-regulation was low in both cultivars across all timepoints, with the most (6 TRGs) occurring in cv. Stigg at 8 dpi accounting for 1.46% of the overall DEGs downregulated. TRG up-regulation was low at 4 dpi but more prevalent at 8 and particularly at 12 dpi. At 12 dpi. Many more TRGs were STB up-regulated in cv. Stigg than in cv. Gallant (48 in common and an additional 173 STB up-regulated in cv. Stigg). Two candidate TRGs were selected for further investigation: due to their original responsiveness to *Z. tritici* infection in the host, they were termed Septoria Responsive Taxonomically Restricted Gene(s) (*TaSRTRG6* and *TaSRTRG7*). They were selected based on their expression profiles (Table 2). *TaSRTRG6* was significantly up-regulated 5.5-fold in cv. Gallant at 4 dpi and 57.1-fold at 12 dpi in cv. Stigg. *TaSRTRG7* was significantly up-regulated 34-fold at 8 dpi in cv. Gallant and 105.7-fold in cv. Stigg at 12 dpi, respectively) (*P* < 0.05).

**TABLE 1:** The transcriptome response of wheat cultivars Stigg and Gallant to Septoria tritici blotch disease, as determined using the Affymetrix *T. aestivum* 61k microarray.

**Regulation^a^**	**Timepoint (dpi)^b^**	**Differentially expressed genes^c,e^**				**Taxonomically restricted genes^d,e^**			
		**Gallant**	**Stigg**	**Common**	**Total**	**Gallant**	**Stigg**	**Common**	**Total**
Down	4	63	6	0	69	1	1	0	2
	8	248	410	30	718	1	6	0	7
	12	746	337	51	1185	0	0	0	0
Up	4	52	46	0	98	1	0	0	1
	8	1202	373	133	1841	47	7	5	59
	12	1503	1352	259	3373	0	173	48	221

*^*a*^Up or down regulated in response to Zymoseptoria tritici isolate IPO323. ^*b*^Days post inoculation with Zymoseptoria tritici. ^*c*^Total number of differentially expressed microarray probes. ^*d*^Pooaceae-specific genes. ^*e*^Genes were either uniquely STB-responsive in either cv. Stigg or Gallant at a given time point, or commonly regulated in both cultivars.*

**TABLE 2:** Fold change for *TaSRTRG6* and *TaSRTRG7* in wheat cultivars Stigg and Gallant in response to Septoria tritici blotch disease, based on Affymetrix *T. aestivum* 61k microarray data.

**Gene^a^**	**Cultivar^b^**	**Timepoint (dpi)^c^**	**Fold-change^d^**	***P-*value**
*TaSRTRG6*	Stigg	4	12.3	0.24
		8	2.0	0.21
		12	57.2	0.01*
	Gallant	4	5.5	0.03*
		8	12.4	0.2
		12	14.4	0.16
*TaSRTRG7*	Stigg	4	9.1	0.24
		8	7.3	0.17
		12	105.7	0.04*
	Gallant	4	2.0	0.61
		8	24.0	0.03*
		12	33.5	0.06

^*a*^Taxonomically restricted gene of interest. ^*b*^STB-resistant cultivar Stigg or STB-susceptible cultivar Gallant. ^*c*^Days post inoculation with Zymoseptoria tritici. ^*d*^Fold-change relative to the Tween20-treated control. *p ≤ 0.05.

### Characterization of TaSRTRG6 and TaSRTRG7

**FIGURE 2:**
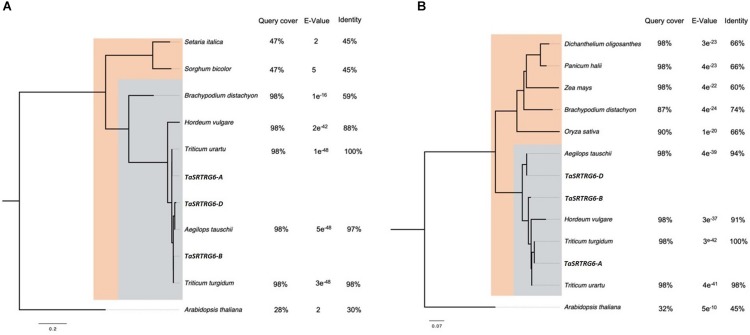
Neighbor-joining phylogenetic trees showing the taxonomic distribution of two taxonomically restricted Septoria responsive genes (TaSRTRGs). Trees are rooted by the top BLASTx hit from the *Arabidopsis thaliana* genome. Orthologs from other species were identified via BLASTx search of the TaSRTRG A homoeolog sequence to the NCBI protein database. **(A)** *TaSRTRG6* is restricted to the Pooideae subfamily (blue box), with weak homology to species outside of the Pooideae but within the Poaceae family (orange box). **(B)** *TaSRTRG7* is found in species from within the Pooideae (blue box) and the Poaceae (orange box). *TaSRTRG7* was not found in any species outside of the Poaceae.

*TaSRTRG6* and *TaSRTRG7* are taxonomically restricted to the Poaceae family (Figure 2). There are three homoeologs of *TaSRTRG6* in the wheat genome: *TaSRTRG6-A* (TraesCS1A 01G265600: A genome), *TaSRTRG6-B* (TraesCS1B01G276500; B genome) and *TaSRTRG6-D* (TraesCS1D01G265800; D genome). Seven paralogs of these genes are also present in the genome: TraesCS1A01G265800, TraesCS1B01G276800, TraesCS1B01G276300, TraesCS1B01G276200, and TraesCS 1D01G266000. *TaSRTRG6* homoeologs are restricted to the core Pooideae subfamily, and are only found within the tribes *Triticeae, Brachypodieae* and *Hordeum*. *TaSRTRG6* genes have high homology to members of the *Triticeae* (*Triticum* spp., *Aegliops tauschii*, and *Hordeum vulgare*) and lower homology to *Brachypodium distachyon*, a member of the Pooideae outside of the *Triticeae* tribe. Outside of the Pooideae, *TaSRTRG6* genes show weak homology to other members of the Poaceae family (*Setaria italica* and *Sorghum bicolor*) (Figure 2). The wheat genome also encodes three homoeologs of TaSRTRG7: *TaSRTRG7-A* (TraesCS3A01G093900.1; A genome), *TaSRTRG7-B* (TraesCS3B01G109200; B-genome) and *TaSRTRG7-D* (TraesCS3D01G094200; D genome). *TaSRTRG7* is distributed throughout the Poaceae family. It is present in all subfamilies except the *Chloidoideae* and is present in all tribes with the exception of the *Aveneae*. Homology to species outside of the Pooideae is considerably lower than to those within the Pooideae (Figure 2).

**FIGURE 3:**
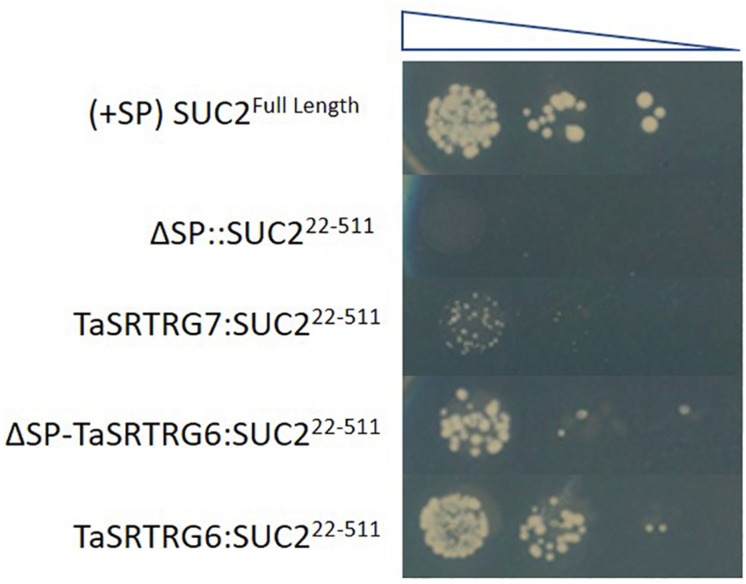
Testing of wheat taxonomically restricted genes (TRGs) secretion using a yeast expression system. The expression of the TaSRTRG6 with a positive secretion signal results in the secretion of the SUC2 protein allowing the growth of yeast transformants on the sucrose-containing media. The TaSRTRG7 transformants without a secretion signal cannot grow on media. (+SP) SUC2^Full Length^ denotes the yeast strain transformed with the full length SUC2 (with signal peptide) gene and served as a positive control while ΔSP:SUC2^22–511^ denotes the yeast strain transformed with the full length SUC2 (without signal peptide) and served as a negative control. TaSRTRG7:SUC2^22–511^ represents the yeast strain transformed with the full length SUC2 (with signal peptide) gene and TaSRTRG7. TaSRTRG6:SUC2^22–511^ represents yeast strain transformed with the full length SUC2 (with signal peptide) gene and TaSRTRG6 while ΔSP-TaSRTRG6:SUC2^22–511^. This experiment included 9 biological replicates (3 × 3 trials) per construct per dilution.

**FIGURE 4:**
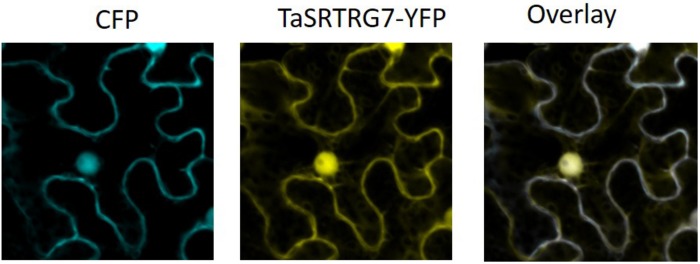
Subcellular localization patterns in *Nicotiana benthamiana*. Agrobacterium-mediated transient co-expression of CFP and TaSRTRG7 -YFP in *N. benthamiana* leaves. CFP denotes cyan fluorescent protein and TaSRTRG7-YFP denotes the TaSRTRG7-yellow fluorescent fusion. This experiment included 9 biological replicates per construct (3 × 3 trials).

Domain analysis indicated that, as expected based on the selection criteria, neither TaSRTRG6 nor TaSRTRG7 encode high confidence conserved domains. TaSRTRG6 did have low homology with a Bowman-Birk type proteinase inhibitor (2.48e^–09^), while TaSRTRG7 had homology with a potato inhibitor domain (6.57e^–23^). An assay investigating the trypsin and chymotrypsin-inhibiting activity of both TaSRTRG6 and TaSRTRG7 was conducted but no significant inhibition was recorded (Supplementary Figure S1). The SignalP 5.0 software (Armenteros et al., 2019) predicted the presence a signal peptide in TaSRTRG6 (0.9996 likelihood) and did not predict the presence of a signal peptide in TaSRTRG7 (0.0008 likelihood). To test the secretion of TaSRTRG6, a complementation assay in which the survival of the host depends on the secretion of the protein of interest was chosen. The results showed that only the TaSRTRG6 protein could complement the suc2 knock out strains (Figure 3). This suggests that the TaSRTRG6 protein is a secreted protein and that TaSRTRG7 is a non-secreted protein. *In planta* localization studies were used to validate the prediction that TaSRTRG7 was non-secreted. Transient expression of YFP-tagged TaSRTRG7 protein was used to determine its subcellular location in tobacco cells using confocal microscopy (Figure 4). TaSRTRG7:YFP and CFP alone were transiently co-expressed in *N. benthamiana* and confocal microscopy was used to observe their localization patterns. Results presented in Figure 4 demonstrate that the TaSRTRG7:YFP fusion had overlapping subcellular localization patterns with CFP in the nucleus and cytoplasm in *N. benthamina* suggesting that it was not secreted into the apoplast. As TaSRTRG6 was predicted and demonstrated to be secreted, no attempts at a localization assay were made as secreted proteins are indistinguishable from membrane-bound proteins *in planta*.

**FIGURE 5:**
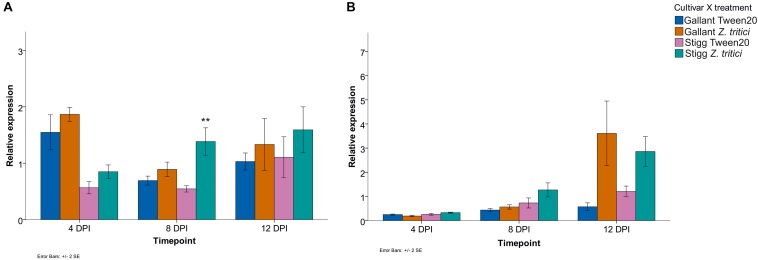
Relative expression of (A) *TaSRTRG6* (A,B) *TaSRTRG7* in the STB susceptible and resistant cvs. Gallant and Stigg in response to inoculation with *Zymoseptoria tritici* isolate Cork Cordiale 4 (at 4, 8, and 12 dpi). Results are based on three independent trials, each including four pooled plant samples per treatment per cultivar per time point combination which were bulked and subsampled twice for RNA extraction (resulting in 3 composite biological × 2 technical replicates per treatment per time point per genotype). Gene expression was assessed at 4, 8, and 12 dpi (days post inoculation) with either a Tween20 or *Z. tritici* application. Y-axis delta CT values are relative expression values of treatments calculated using the 2^(–ΔΔCt)^ formula using the average of two housekeeper genes GAPDH and Alpha tubulin. Bars indicate SEM. **p ≤ 0.01.

Additional STB seedling experiments were conducted to determine if the genes were responsive to an aggressive field isolate (Cork Cordiale 4) of *Z. tritici* in wheat cvs. Gallant and Stigg, using primers that target all three homologs of either *TaSRTRG6* or *TaSRTRG7*. Based on the microarray analysis, *TaSRTRG6* was upregulated 5.5-fold in cv. Gallant at 4 dpi and upregulated 57.1-fold at 12 dpi in cv. Stigg in response to isolate IPO323. qRT-PCR validated that this gene was STB up-regulated in cv. Stigg at 8 dpi in response to Cork Cordiale 4 (*P* = 0.008), but in cv. Gallant the increases in *TaSRTRG6* gene expression due to STB disease at 4, 8, or 12 dpi were not statistically significant (*P* = 1, 1, and 0.640 respectively) (Figure 5A). Furthermore, the basal expression was higher in cv. Gallant as compared to cv. Stigg at 4 dpi. According to the microarray analysis, *TaSRTRG7* was upregulated 34-fold at 8 dpi in cv. Gallant and 105.7-fold in cv. Stigg at 12 dpi in response to isolate IPO323. Although the qRT-PCR analysis indicated that there was a 5.5- and 2-fold change in this gene at 12 dpi in cvs. Gallant and Stigg in response to isolate Cork Cordiale 4, differences were not significant due to the high variance in expression (*P* = 0.068 and 0.785 respectively*;* Figure 5B). A pattern of accumulation was observed for both *TaSRTRG* genes in cv. Stigg while this was only observed in *TaSRTRG7* in cv. Gallant. Overall expression levels between both cultivars was comparable with like-for-like treatments (Figure 5). *In silico* analysis of RNAseq data also further validated the responsiveness of these genes to STB (isolate IPO323) in the susceptible cv. Sevin (Yang et al., 2013; Supplementary Figure S2). This analysis also unveiled their responsiveness to other pathogens, including *F. graminearum, F. pseudograminearum*, *B. graminis*, and the PAMP-elicitors chitin and Flg22 (Kugler et al., 2013; Zhang et al., 2014; Powell et al., 2017; Ramirez-gonzalez et al., 2018).

### Virus Induced Gene Silencing

**FIGURE 6:**
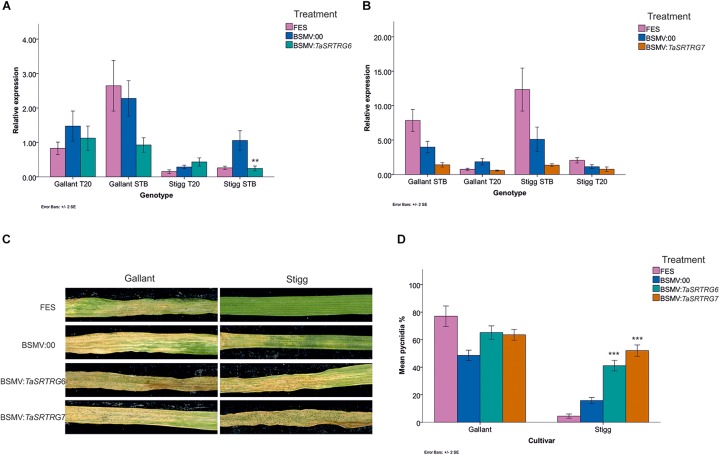
The impact of virus-induced gene silencing of *TaSRTRG6* and *TaSRTRG7* on the development of Septoria tritici blotch disease in the STB resistant and susceptible cultivars Stigg and Gallant. Viral RNA was applied to the second emergent seedling leaves and gene expression was quantified in the third leaves, while disease was assessed in the fourth leaves. This experiment included three trials, each with four independent replicates per treatment (12 biological replicates per treatment). **(A,B)** Analysis of the efficacy of VIGS of *TaSRTRG6* and *TaSRTRG7*, respectively, 12 days post inoculation with *Z. tritici* isolate Cork Cordiale 4 (19 days post inoculation with BSMV). Relative expression was calculated using the 2^(–ΔΔCt)^ formula, using the average of the two reference genes GAPDH and Alpha tubulin. FES is the VIGS application buffer; BSMV:00 is the empty vector control and BSMV:TaSRTRG6 and BSMV:TaSRTRG7 denote the silencing treatments for *TaSRTRG6* and *TaSRTRG7*, respectively. Significance denoted by a double asterisk denotes a *P*-value of ≤ 0.01 as compared to its respective BSMV:00 control treatment of the same genotype. Bars indicate SEM. **(C)** Visualization of the effect of VIGS on STB disease development at 21 days post inoculation with *Z. tritici*. **(D)** Quantification of the effect of VIGS on STB disease development (diseased leaf area bearing pycnidia) on wheat cultivars Gallant (left) and Stigg (right) at 21 dpi. Asterisks (***) signify *P* ≤ 0.001 as compared to the BSMV:00 control (blue bar) within a given genotype cluster. Bars indicate SEM.

VIGS fragments were designed to silence all homeologs of either *TaSRTRG6* or *TaSRTRG7*, using 129 and 95 bp fragments, respectively, in seedlings of wheat cvs. Stigg and Gallant. A 185bp fragment of the barley phytoene desaturase (PDS) was used as a positive control, as described by Scofield et al. (2005). The efficacy of VIGS applied to the second leaf in silencing gene expression in the third leaf was confirmed using gene-specific qRT-PCR analysis, relative to the effect of control BSMV:00 treatment (at 19 days post VIGS treatment and 12 dpi with *Z. tritici* isolate Cork Cordiale 4). *Z. tritici* treatment activated both genes in cvs. Stigg and Gallant, although differences were not always significant at *P* < 0.05 (Figures 6A,B). In cvs. Gallant and Stigg, the *Z. tritici*-treated BSMV:*TaSRTRG6* treatment resulted in a 1.7 and 4.5-fold reduction, respectively, in *TaSRTRG6* gene expression, relative to BSMV:00, with the latter reduction being statistically significant (*P* < 0.05; Figure 6A). For *TaSRTRG7* silencing (Figure 6B), cvs. Gallant and Stigg respectively showed reductions of 2.8 and 3.8-fold in *TaSRTRG7* transcript levels in *BSMV:TaSRTRG7* relative to BSMV:00 treatment, although the high variation meant that reductions were not significant at *P* < 0.05.

The fourth leaf of VIGS-treated plants was phenotyped for STB disease development at 21 dpi with *Z. tritici*, based on the percentage of disease leaf area bearing pycnidia. Symptom expression was high on cv. Gallant (77% on FES and 49% on BSMV:00) and *TaSRTRG6* and *TaSRTRG7* silencing, relative to BSMV:00 treatment, caused a small (1.3-fold) but insignificant (*P* > 0.054) increase in disease levels (Figure 6D). However, silencing of both genes significantly enhanced the susceptibility of cv. Stigg to STB disease (by 3–5-fold, relative to BSMV:00: *P* = 0.0000; Figure 6D). In the resistant cv. Stigg, the average observed percentage diseased leaf area bearing pycnidia was 5% in the FES treatment and 9% in the BSMV*:*00 empty vector treatment. BSMV:TaSRTRG6 plants exhibited an average score of 41% coverage and BSMV:TaSRTRG7 exhibited an average score of 52%. Silencing of BSMV:TaSRTRG7 in the resistant cv. Stigg led to similar disease levels that were observed in the BSMV:00 treatment of the susceptible cv. Gallant. Thus, we concluded that silencing of both *TaSRTRG6* and *TaSRTRG7* reduced host resistance to STB in the resistant variety but did not appear to greatly induce hyper-susceptibility in the susceptible host. The VIGS control treatment BSMV:PDS resulted in the expected photobleaching of leaves (results not shown).

### TaSRTRG Genes Interact With *Z. tritici* SSPs

**FIGURE 7:**
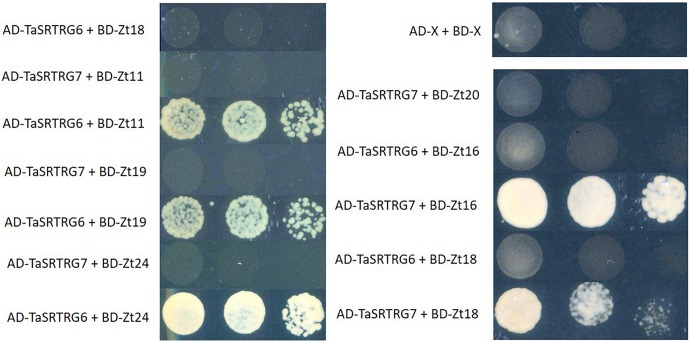
Yeast two-hybrid evaluation of the interaction between TaSRTRG proteins and small secreted (Zt) proteins from *Zymoseptoria tritici.* Matchmaker Gold yeast strains carrying the bait vector pGADT7 containing *TaSRTRG6* or *TaSRTRG7* were transformed with the prey vector pGBKT7 containing *Z. tritici* small secreted Zt proteins. This experiment included 9 biological replicates per construct combination (3 × 3 trials). Yeast spotting on the media was performed using serial dilutions from an initial OD600 of 0.1, 0.01, and 0.001, respectively, and strains were spotted on synthetic defined selective media (lacking leucine, tryptophan, histidine, and adenine, -LTHA) and incubated at 30°C for 3 days. AD denotes the activating domain and BD denotes the binding domain.

**FIGURE 8:**
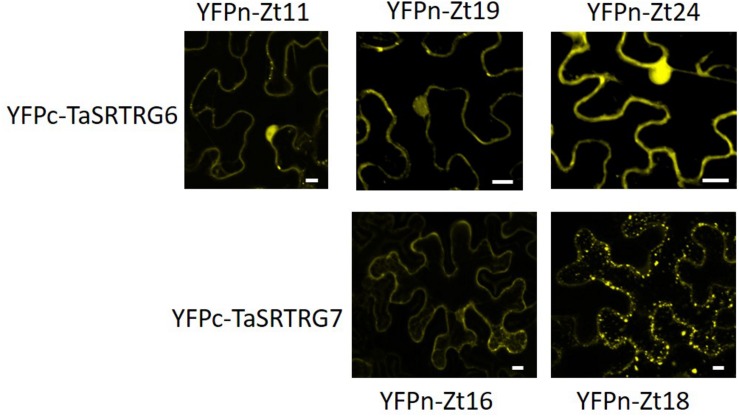
Bimolecular fluorescence complementation analysis of the interactions between TaSRTRG proteins and *Zymoseptoria tritici* small secreted Zt proteins, as visualized in *N. benthamiana*. Leaves were infiltrated with an equal mixture of both *A. tumefaciens* strain expressing YFPn-Zt proteins or YFPc-*TaSRTRGs* fusion proteins. A total of 4 biological replicates were tested per SSP infiltration in each of 3 independent trials (12 biological replicates per construct combination). Leaf epidermal cells were imaged after 48 h by confocal microscopy. Bars: 10 μm.

A library of 27 potential *Z. tritici* SSPs (Karki, unpublished) were tested for their ability to interact with either TaSRTRG6 or TaSRTRG7 using Y2H and BiFC. Using a galactose-responsive transcription factor GAL4 (GAL4)-based Y2H system, we deduced that TaSRTRG6 interacted with three of 27 *Z. tritici* SSPs (Zt11, Zt19, and Zt24) and TaSRTRG7 interacted with two of the 27 *Z. tritici* SSPs tested (Zt16 and Zt18) (Figure 7). No common SSP was seen to interact with both TaSRTRG6 and TaSRTRG7. BiFC analysis was used to analyze TaSRTRG and SSP interactions *in planta* (in tobacco). For BiFC analysis, Cnx6 homodimerization *in planta* was used as a positive control which gave a clear fluorescence. As Zt18 did not interact with TaSRTRG6 and Zt11 did not interact with TaSRTRG7, these were used as respective negative controls wherein no fluorescence was observed (Supplementary Figure S3). In the BiFC assay, the N terminal part of the YFP was fused to the N-terminal of TaSRTRG6 and TaSRTRG7 (without signal peptide) to create YFPn-TaSRTRG6 and YFPn-TaSRTRG7. The C terminal part of YFP was fused to the N-terminal of *Z. tritici* SSPs without signal peptide, to create YFPc-*Z. tritici* SSP fusions. Interactions between these wheat/*Z. tritici* fusion proteins were then tested by *Agrobacterium*-mediated transient coexpression in *N. benthamiana*. A strong YFP signal in the cytoplasm of leaf cells co-infiltrated with *A. tumefaciens* was observed after 48 h (Figure 8). TaSRTRG6 was observed interacting with three separate *Z. tritici* SSPs, namely Zt11, Zt19, and Zt24. TaSRTRG7 was observed interacting with two *Z. tritici* SSPs, namely Zt16 and Zt18. The secretory signals were removed from proteins for BiFC and thus localization is irrelevant. Non-ethless, it is interesting to note that several interactions occur in the nucleus and several occurred in punctate bodies (Figure 8). There was no common SSP that interacted with both wheat proteins, at least in *N. benthamiana.* Both positive and negative controls behaved as expected, thus, the BiFC data validated that both TaSRTRG6 and TaSRTRG7 can physically interact *in planta* with *Z. tritici* SSPs.

## Discussion

This study validated the role of some rapidly evolving wheat genes, which in this instance, serve as an important component of the response to STB disease. They were identified from a pipeline extracted that was used to extract genes from microarray data. Although we successfully identified TRGs from this data, we hypothesize that mining RNAseq data for TRGs may yield higher numbers of TRGs, and more species specific TRGs (rather than those that are family or subfamily specific). This is due to microarray probes often being designed based on expressed sequence tags and UniGene sequences (Affymetrix wheat 61K array data sheet, 2020), and therefore may not be inclusive of all wheat genes, rare variants and/or species/cultivar specific transcripts. Additionally, the wheat 61k microarray contains only ∼61,000 probes, whereas the most recent reference annotation of *T. aestivum* contains 133,346 coding sequences (IWGSC, 2018). Therefore, using RNAseq for the identification of species-specific TRGs will almost certainly provide more extensive coverage of the most recently evolved and taxonomically restricted genes within the wheat genome.

At present the discovery of TRGs is hampered by their absence within current genome assemblies that primarily represent conserved genes, although the inclusion of low confidence genes in pipelines will unearth many more interesting genes. Most Septoria responsive TRGs were not common to both wheat cultivars and a higher number of STB-responsive TRGs were present in the susceptible cv. Gallant than the resistant cv. Stigg from both an upregulation and downregulation standpoint). This difference is likely not due to cv. Gallant possessing more STB-responsive TRGs, but due to relatively quick colonization of cv. Gallant as opposed to cv. Stigg by *Z. tritici* (as reflected in the total microarray transcriptome response shown in Supplementary Table S3). Based on field trials conducted in Ireland, the latent phase duration of cvs. Gallant and Stigg was recorded as 19 and 29 dpi, respectively (Hehir et al., 2017). The extensive latent phase exhibited by cv. Stigg suggests that the fungus enters the necrotrophic phase much later than it does in cv. Gallant and as a result a reduced amount of active defense pathways and ultimately associated TRGs would in theory be active at the timepoints investigated. Although the delineation of STB-responsive TRGs was the primary focus of this study, the microarray data also revealed the differences that occur between resistant and susceptible cultivars during *Z. tritici* infection (see Supplementary Table S3). From the microarray, a total of 290 unique probes that were taxonomically restricted were expressed. With the exception of cv. Stigg downregulation at 8 dpi, cv. Gallant had consistently higher numbers of DEGs in both up and downregulation at all timepoints. Interestingly, cv. Stigg had more unique TRGs downregulated at 8 dpi and upregulated at 12 dpi as compared to the susceptible cultivar, accounting for a substantial portion cv. Stigg’s response to infection in the latter instance. This TRG enrichment is possibly owing to Stigg’s *Triticum turgidum* subspecies *dicoccoides* heritage. If the fungus requires (or can manipulate) host defense processes for disease progression, as suggested by Rudd et al. (2008), a torpid response of genes typically associated with defense in cv. Stigg could lead to the lengthy latent phase that has been observed in the field (Hehir et al., 2017) and with multiple *Z. tritici* isolates under glasshouse conditions (Rahman et al., 2020). It is possible that cv. Gallant is employing a classical defense strategy or is being manipulated into doing so, while cv. Stigg has a more specialized approach or is less prone to manipulation by the pathogen. cv. Stigg has QTL for STB resistance on various chromosomes for chlorosis, (3DL and 2BL) necrosis (3DL, 1BS, and 2BL) and pycnidia (3DL, 1BS, and 2BL) (Odilbekov et al., 2019). Interestingly, homeologs of both *TaSRTRG6* and *TaSRTRG7* were found on chromosomes 1B and 3D respectively, but neither were located within the QTL intervals on either chromosome.

Two *TaSRTRG* genes, *TaSRTRG6* and *TaSRTRG7* were targeted for further study based on their relatively high expression (>20-fold increase) in the microarray in response to *Z. tritici* isolate IPO323. Another experiment validated that the genes respond to the more aggressive isolate of *Z. tritici:* Cork Cordiale 4, albeit differences were not always significant and TaSRTRG6 expression peaked earlier than that of TaSRTRG7, particularly in the susceptible cultivar. Inhibition assays did not validate predictions of protease inhibitor activity within either TaSRTRG6 or TaSRTRG7. Unlike TaSRTRG7, TaSRTRG6 was secreted into the apoplast, and both TaSRTRG proteins interacted with a distinct set of fungal SSPs. It seems likely therefore that TaSRTRG6 might serve a more active role during the latent phase, perhaps being secreted into the apoplast to directly counter fungal SSPs secreted during establishment, while TaSRTRG7 may be more active during later stages of infection when the pathogen is transitioning into to a necrotrophic stage and interacting with a separate suite of fungal SSPs. Unfortunately, at this time, the temporal expression of the fungal SSPs has not been investigated but may be in future studies. This hypothesis was also supported by the expression profile of *TaSRTRG6* and *TaSRTRG7* in response to mildew, as determined by *in silico* analysis of gene expression data (Borrill et al., 2016). *TaSRTRG7* was response to mildew at 48 hpi while *TaSRTRG6* was responsive at 24 hpi. Generally, 24 hpi coincides with haustorium penetration during the wheat/mildew interaction and may suggest that *TaSRTRG6* plays a role in early response or recognition of certain fungal pathogens or their SSPs in an extracellular capacity, while *TaSRTRG7* may serve an internal or downstream role as it was primarily responsive after an additional 24 h had elapsed.

VIGS validated that both TaSRTRG6 and TaSRTRG7 contribute to STB resistance. Silencing all homoeologs of both *TaSRTRG* genes rendered cv. Stigg highly susceptible to STB disease. Effects on the susceptibility of cv. Gallant were much more muted but may have been masked by its’ inherent susceptibility to STB disease. What is obvious is that these gene products are central to one or more components of resistance in cv. Stigg. cv. Stigg is derived from *Triticum turgidum* subspecies *dicoccoides*. It has introgressions from *dicoccoides* on the both the A and B genome and also possesses *Z. tritici* resistance QTLs on the 3DL, 2BL, and 1BS chromosomes. While the resistance observed in cv. Stigg may be due to the presence of resistance QTLs, the robustness of this observed in the field does not necessarily fit with qualitative resistance models wherein a presence/absence phenotype can be observed depending on the cultivar/isolate combination. Instead, cv. Stigg has shown resistance to a diverse range of field isolates wherein disease symptoms manifest eventually, albeit on a comparatively protracted timescale as compared to cv. Gallant (Rahman et al., in review). The introgression of *Triticum turgidum* subspecies *dicoccoides* in the pedigree of cv. Stigg may be a factor in the compatibility of the pathogen, perhaps making cv. Stigg less receptive to a highly tuned repertoire of manipulative SSPs.

*TaSRTRG6* is not a species-specific TRG and given its presence in the *Brachypodieae* and *Hordeum* it is safe to assert that its emergence occurred before the domestication of wheat, however, its divergence from the other members of the Poaceae suggests specialization. The interactions that occurred between TaSRTRG6 and the fungal SSPs would likely take place outside of the host cells given both the secretion signals of TaSRTRG6 and *Z. tritici*’s penchant for apoplastic habitation. The phenotypic effect of the silencing of this gene coupled with its taxonomical classification of taxonomically-restricted gene and potential involvement in other fungal pathogen responses suggests that this protein may have a broader role in the general response to fungal pathogen attacks by interfering with or disrupting SSPs that are secreted during infection. At this juncture, comparatively little is known about the three *Z. tritici* SSPs (Zt11, Zt19, and Zt24) that interacted with TaSRTRG6, save that Zt11 and Zt19 induced cell death in the non-host *N. benthamiana* (Supplementary Figure S4). This cell death induction may translate to the true host – wheat, but at this time has not been shown. Nevertheless, the cell death phenotype does prove that these fungal proteins can affect plant survival processes. These SSPs may have a function in interfering with host defenses, masking fungal establishment or even triggering the necrotrophic phase of infection but at this moment too little is known about their function other than that when operating in the absence of *TaSRTRG6*, disease severity is increased in the resistant cultivar.

TaSRTRG7 is distributed throughout the Poaceae family. It is present in all subfamilies except the Chloidoideae and is present in all tribes with the exception of the Aveneae. TaSRTRG7 interacted with two fungal SSPs (namely Zt16 and Zt18), of which Zt18 was capable of inducing cell death in *N. benthamiana* (Supplementary Figure S4). Interestingly however, TaSRTRG7 does not appear to be secreted into the apoplast and thus suggests that in order for the protein interaction to be effective in their interacting capacity, the fungal SSPs in question must enter the host cell. Integration of pathogenic fungal SSPs into the host cytoplasm has been documented in other grasses (Khang et al., 2010), and could be part of the latter or transitional stages of *Z. tritici* infection.

In conclusion, this study has demonstrated that through the use of a mining pipeline, taxonomically restricted genes can be selected, and their functions explored to better understand specific plant/pathogen interactions. The roles of taxonomically restricted genes should not be overlooked and their interactions with fungal SSPs highlight their specialization in a silent conflict that has been ongoing for over 10,000 years. While this study was specific to the *Z. tritici*/wheat interaction, modified mining pipelines could be used to extract TRGs responsive to a numerous biotic and abiotic stresses governed by the differential stresses imposed on source material. Building on the concepts established during this study, mining for taxonomically restricted genes from *de novo* assemblies would allow for the discovery and study of TRGs that may be exclusively specialized for combatting *Z. tritici*. Understanding the interactions that occur between a broad selection of *Z. tritici* SSPs active in the field and the various resistance genes present in contemporary varieties could allow for more precise methods of control and a deeper understanding of this specialized host/pathogen interaction. Future studies will seek to unravel the role of TaSRTRG6 and TaSRTRG7 in STB resistance.

## Data Availability Statement

The datasets generated for this study can be found in the Figshare https://doi.org/10.6084/m9.figshare.11882601.v1.

## Author Contributions

CB, BZ, EM, and FD designed the experiment. AO’D conducted the microarray experiment. BZ, CB, JH, SA, and AO’D carried out all experiments. CB and HB analyzed the data. SK and AF identified SSPs from *Z. tritici*. CB and BZ wrote the manuscript. HB, EM, and FD reviewed the manuscript.

## Conflict of Interest

The authors declare that the research was conducted in the absence of any commercial or financial relationships that could be construed as a potential conflict of interest.

## Footnotes

1 www.affymetrix.com

2 http://www.plexdb.org/

3 https://urgi.versailles.inra.fr/download/iwgsc/IWGSC_RefSeq_Annotations/v1.1/

4 https://www.ncbi.nlm.nih.gov/

5 http://tree.bio.ed.ac.uk/software/figtree/

6 http://simgene.com/Primer3

7 http://www.cbs.dtu.dk/services/SignalP/
