# Supplementary figures and images for: Taxonomically Restricted Wheat Genes Interact With Small Secreted Fungal Proteins and Enhance Resistance to Septoria Tritici Blotch Disease
Source: Front Plant Sci. 2020 May 7;11:433. doi: 10.3389/fpls.2020.00433 (PMC7236048; doi:10.3389/fpls.2020.00433)

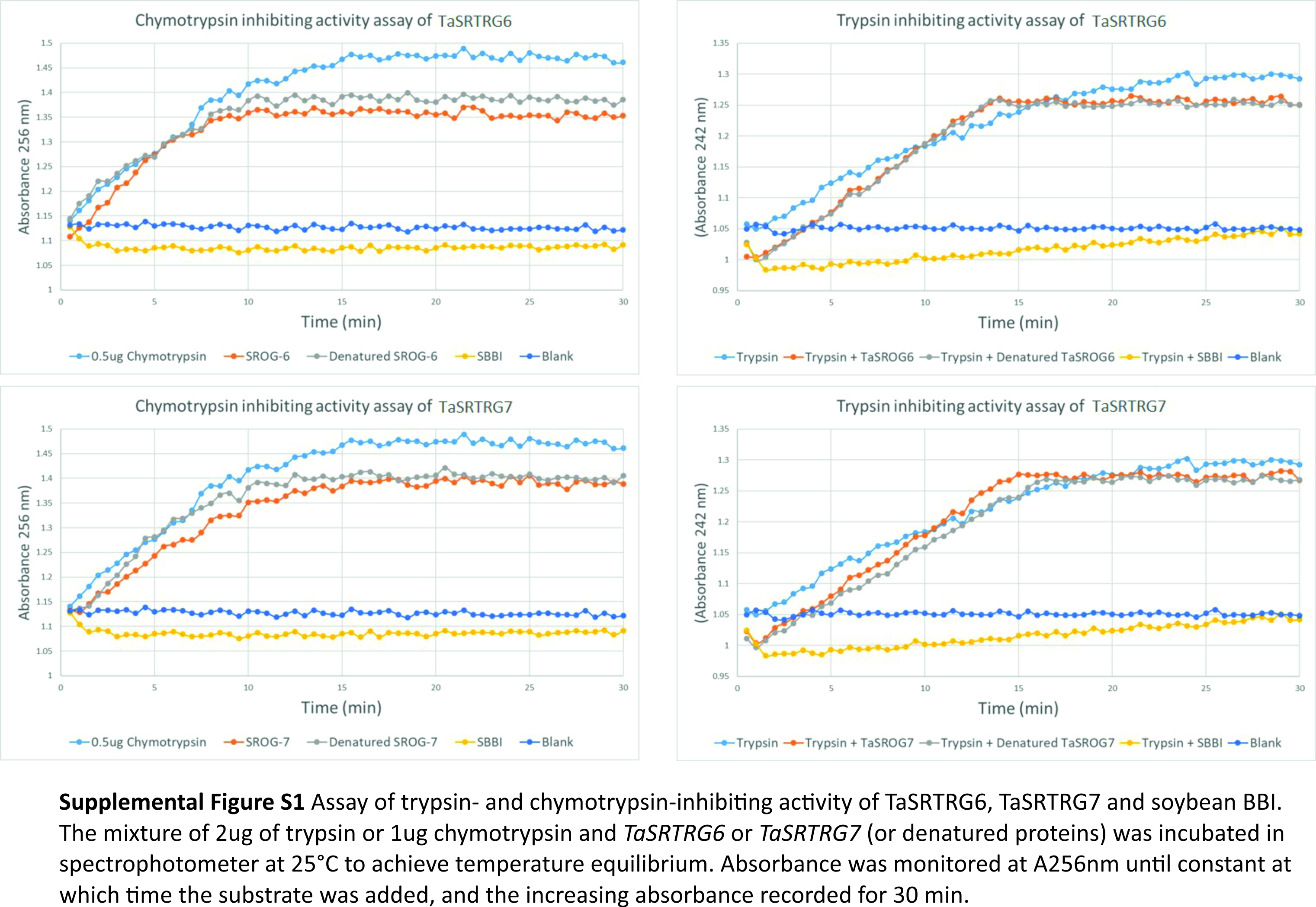

Supplement: Supplementary file 1 [file Image_1.jpeg]

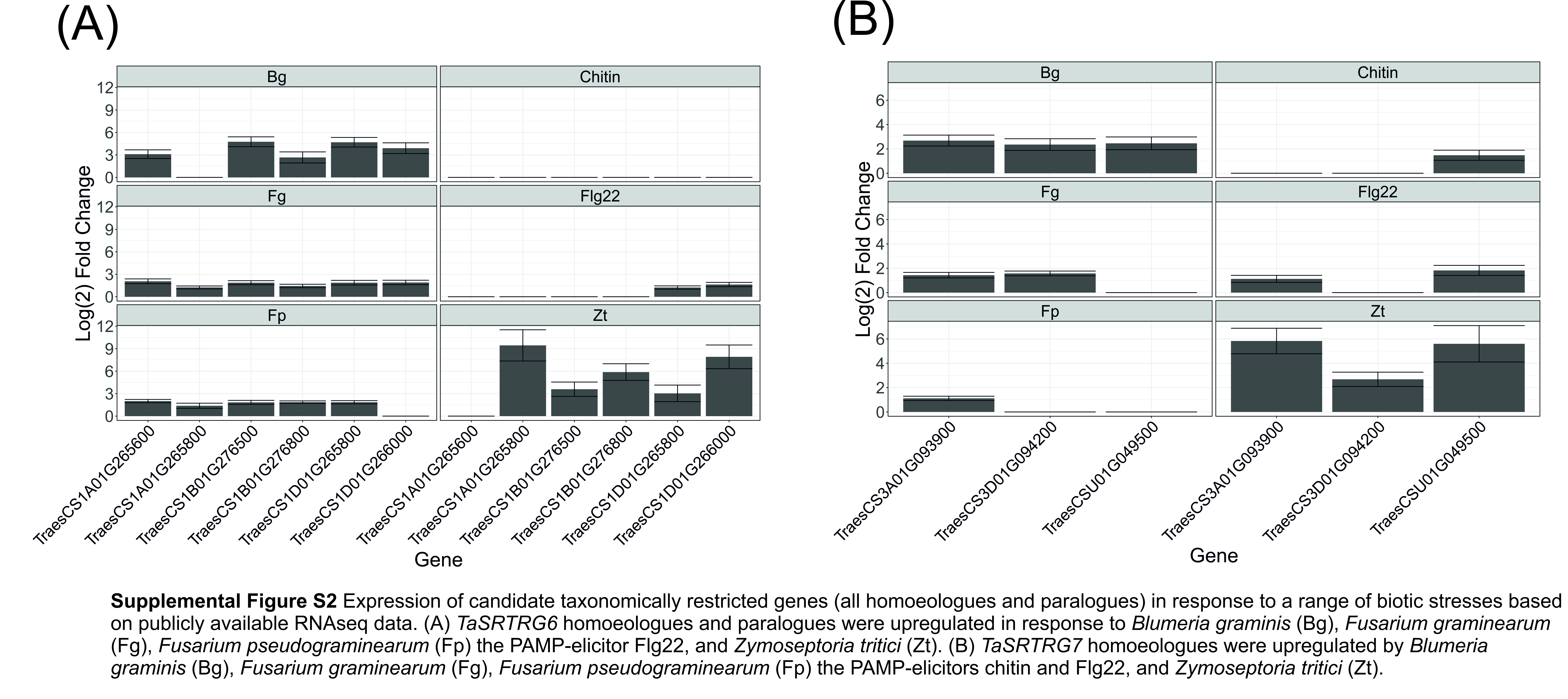

Supplement: Supplementary file 2 [file Image_2.jpeg]

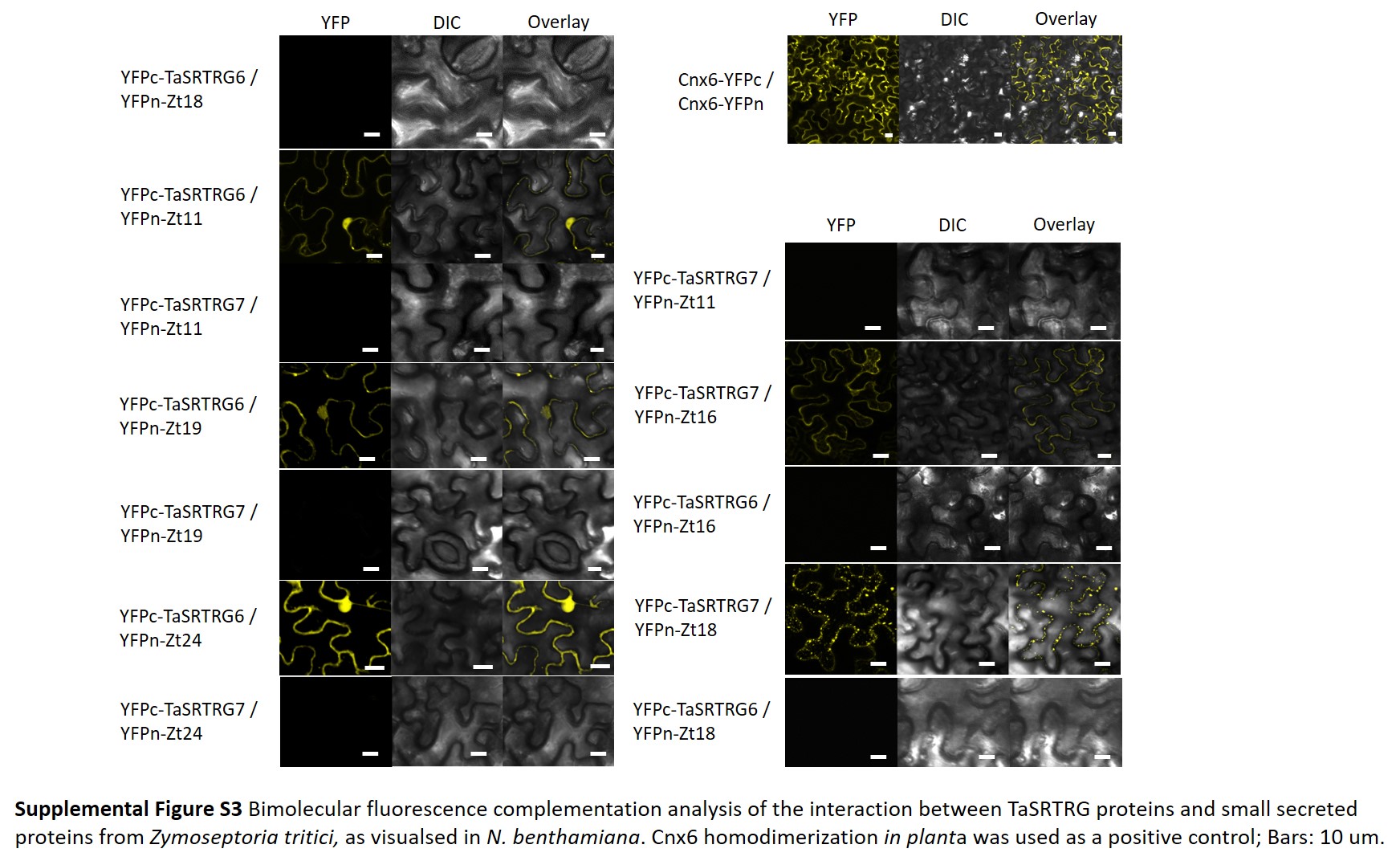

Supplement: Supplementary file 3 [file Image_3.jpeg]

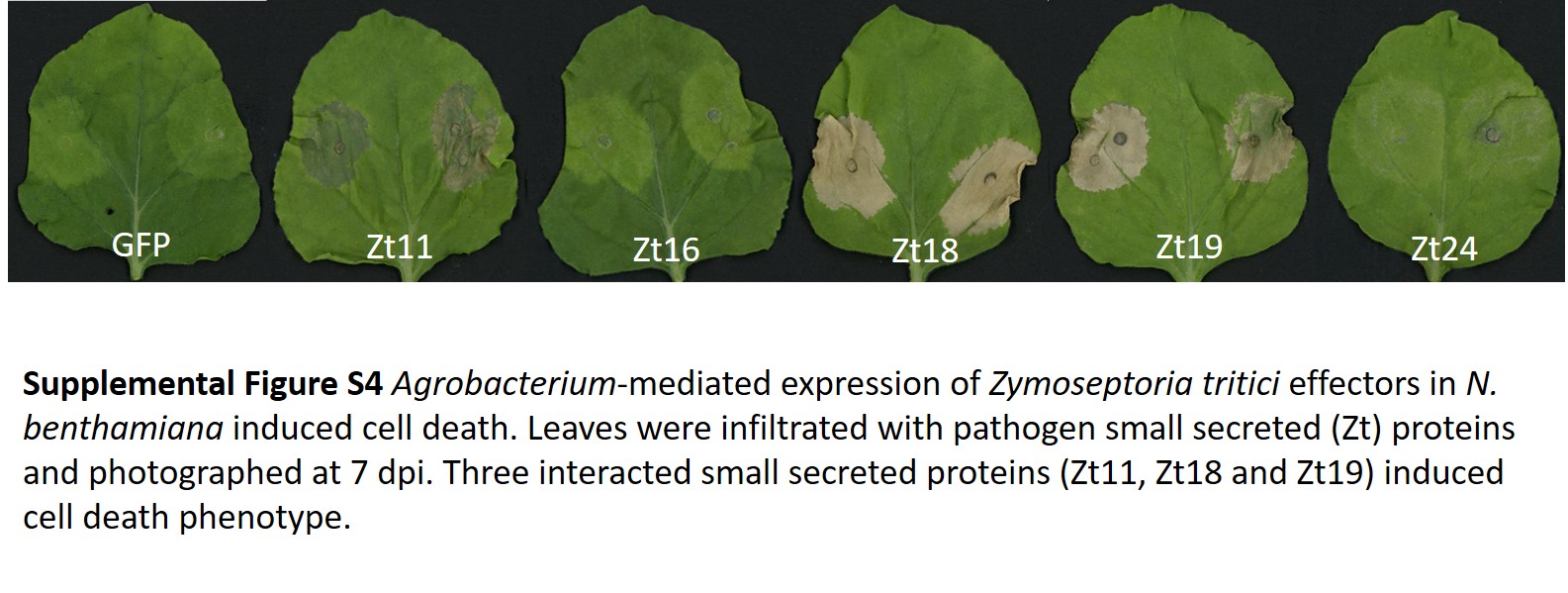

Supplement: Supplementary file 4 [file Image_4.jpeg]
