# Supplementary material for: Taxonomically Restricted Wheat Genes Interact With Small Secreted Fungal Proteins and Enhance Resistance to Septoria Tritici Blotch Disease
Source: Front Plant Sci. 2020 May 7;11:433. doi: 10.3389/fpls.2020.00433 (PMC7236048; doi:10.3389/fpls.2020.00433)
Supplement: Supplementary file 5 [file Table_1.docx]

**Table S1** Primers used in this study.

| **Primer** | **Primer sequence (5' to 3')** | **Application** |
| --- | --- | --- |
| TaSRTRG6-F | ATACCAGTATGTCGTCTCTTCGTC | qRT-PCR and VIGS validation of TaSRTRG6 |
| TaSRTRG6-R | TTATTCACGGAGGATATTCCGG |  |
| TaSRTRG7-F | GCCTGTCCTTCATGATGATCT | qRT-PCR and VIGS validation of TaSRTRG7 |
| TaSRTRG7-R | TACGAGAATGAGCTCCTCGG |  |
| Alpha tubulin | ATCTCCAACTCCACCAGTGT | qRT-PCR and VIGS validation reference gene |
| Alpha tubulin | TCATCGCCCTCATCACCGTC |  |
| Glyceraldehyde phosphate dehydrogenase 2 | TCACCACCGACTACATGA | qRT-PCR and VIGS validation reference gene |
| Glyceraldehyde phosphate dehydrogenase 2 | ACAGCAACCTCCTTCTCA |  |
| TaSRTRG6-VIGS-F | CGATTAATTAACCCCAAGTGCTGCAACAA | Cloning of VIGS fragment BSMV:TaSRTRG6 |
| TaSRTRG6-VIGS-R | CGAGCGGCCGCGCAGCGCCACATTTCGGGGC |  |
| TaSRTRG7-VIGS-F | CGATTAATTAATGCGGGGGATCTCCGCAACG | Cloning of VIGS fragment BSMV:TaSRTRG7 |
| TaSRTRG7-VIGS-R | CGAGCGGCCGCCATCGAGGTCTTCCCGGTCG |  |
| pGEM-T-SP6-F | TATTTAGGTGACACTATAG | pGEM-T vector |
| pGEM-T-SP6-R | CTATAGTGTCACCTAAATA |  |
| pGEM-T-T7-F | TAATACGACTCACTATAGGG |  |
| pGEM-T-T7-R | CCCTATAGTGAGTCGTATTA |  |
| AttB-dSP-ΔSP-TaTRG6-F | GGAGATAGAACCATGGACTTCTTCCCCAAGTGCTG | Cloning TaSRTRG6 without signal peptide into pDONR207 |
| AttB-TaSRTRG6-F | GGAGATAGAACCATGAAGGGCACCAAGCTCGCGGCGATC | Cloning TaSRTRG6 into pDONR207 |
| AttB-TaSRTRG6-R | CAAGAAAGCTGGGTCGTACTTCTTGCAGGGTCC |  |
| AttB-TaSRTRG6-Stop-R | CAAGAAAGCTGGGTCTCAGTACTTCTTGCAGGGTCC |  |
| AttB-TaSRTRG7-F | GGAGATAGAACCATGAGCTCCTCGGACGACAC | Cloning TaSRTRG7 into pDONR207 |
| AttB-TaSRTRG7-R | CAAGAAAGCTGGGTCGCCAATGCGGGGGATCTC |  |
| AttB-TaSRTRG7-Stop-R | CAAGAAAGCTGGGTCCTAGCCAATGCGGGGGATCTC |  |
| attB1 | GGGGACAAGTTTGTACAAAAAAGCAGGCTTCGAAGGAGATAGAACCATG | attB extension for subcloning into pDONR207 |
| attB2 | GGGGACCACTTTGTACAAGAAAGCTGGGTC |  |
| P45-BamH1-ΔSP-TaSRTRG6 | GCGGATCCCGACTTCTTCCCCAAG | Cloning TaSRTRG6 into pET45b+ expression vector |
| P45-Xho1-dSP-TaSRTRG6 | CCGCTCGAGTCAGTACTTCTTGCA |  |
| P45-BamH1-TaSRTRG7 | GCGGATCCCATGAGCTCCTCGGAC | Cloning TaSRTRG7 into pET45b+ expression vector |
| P45-Xho1-TaSRTRG7 | CCGCTCGAGCTAGCCAATGCGGGG |  |
