# Supplementary material for: Taxonomically Restricted Wheat Genes Interact With Small Secreted Fungal Proteins and Enhance Resistance to Septoria Tritici Blotch Disease
Source: Front Plant Sci. 2020 May 7;11:433. doi: 10.3389/fpls.2020.00433 (PMC7236048; doi:10.3389/fpls.2020.00433)
Supplement: Supplementary file 6 [file Table_2.docx]

**Supplemental Table S2** Homology of VIGS constructs to wheat TaSRTRG6 and TaSRTRG7 homeolog gene sequences from cv. Chinese Spring.

| **VIGS construct** | **Gene target** | **Homology of VIGS construct (%)** |
| --- | --- | --- |
| BSMV:*TaSRTRG6* | TraesCS1A02G265600 | 97.7 |
|  | TraesCS1A02G265800 | 95.1 |
|  | TraesCS1B02G276500 | 99.2 |
|  | TraesCS1B02G276800 | 95.1 |
|  | TraesCS1B02G276800 | 95.1 |
|  | TraesCS1D02G266000 | 94.7 |
| BSMV:*TaSRTRG7* | TraesCS3A02G093900 | 100 |
|  | raesCS3D02G094200 | 93.5 |
|  | TraesCSU02G049500 | 93.5 |
